# Near-atomic, non-icosahedrally averaged structure of giant virus Paramecium bursaria chlorella virus 1

**DOI:** 10.1038/s41467-022-34218-4

**Published:** 2022-10-29

**Authors:** Qianqian Shao, Irina V. Agarkova, Eric A. Noel, David D. Dunigan, Yunshu Liu, Aohan Wang, Mingcheng Guo, Linlin Xie, Xinyue Zhao, Michael G. Rossmann, James L. Van Etten, Thomas Klose, Qianglin Fang

**Affiliations:** 1 Scholl of Public Health (Shenzhen), Sun Yat-sen University, Shenzhen, Guangdong 518107 China; 2 Department of Plant Pathology and Nebraska Center for Virology, University of Nebraska-Lincoln, Lincoln, NE 68583-0900 USA; 3 Department of Biological Sciences, Purdue University, West Lafayette, IN 47907 USA

**Keywords:** Cryoelectron microscopy, Virus structures

## Abstract

Giant viruses are a large group of viruses that infect many eukaryotes. Although components that do not obey the overall icosahedral symmetry of their capsids have been observed and found to play critical roles in the viral life cycles, identities and high-resolution structures of these components remain unknown. Here, by determining a near-atomic-resolution, five-fold averaged structure of Paramecium bursaria chlorella virus 1, we unexpectedly found the viral capsid possesses up to five major capsid protein variants and a penton protein variant. These variants create varied capsid microenvironments for the associations of fibers, a vesicle, and previously unresolved minor capsid proteins. Our structure reveals the identities and atomic models of the capsid components that do not obey the overall icosahedral symmetry and leads to a model for how these components are assembled and initiate capsid assembly, and this model might be applicable to many other giant viruses.

## Abstract (web summary)

Giant viruses are large DNA viruses with capsid diameters up to 500 nm. Given technical challenges only few high-resolution structures of viral capsids exist. Here, Shao et al. provide a nearatomic structure of giant virus PBCV-1 capsid and resolve the previously unknown asymmetric capsid components. The local capsid microenvironments created by these components provide insights into the mechanism of capsid assembly of giant viruses.

## Introduction

Giant viruses (or “nucleocytoplasmic large DNA viruses (NCLDVs)”) include Mimiviruses, Iridoviruses, Asfarviruses, Ascoviruses, Marseilleviruses, Pandoraviruses, Poxviruses, and Phycodnaviruses, now known as the *Nucleocytoviricota* (Phylum). These viruses infect a wide range of eukaryotic hosts such as algae, amoebae, insects, fishes, birds, reptiles, mammals, and humans^1–3^, and some are defined by their very large icosahedron-shaped capsids (diameters: 150–500 nm).

The icosahedron-shaped NCLDVs have a roughly icosahedral outer capsid composed of hundreds of pseudo-hexameric capsomers in the icosahedral faces and one pentameric capsomer at each fivefold vertex^4–13^. Each pentameric and pseudo-hexameric capsomer is composed of five copies of a penton protein^11–13^ and three copies of a major capsid protein (MCP), respectively^6,11–13^. These capsomers are arranged into 20 triangular arrays named trisymmetrons and 12 pentagonal arrays named pentasymmetrons, with neighboring symmetrons separated by cleavage lines^14^. Immediately internal to the outer capsid shell is a layer of minor capsid proteins that stabilizes the outer capsid^5,6,11–13^. In addition, capsid components that do not follow the overall icosahedral symmetry (hereafter “asymmetric capsid components”) were observed in some NCLDV capsids. For example, *Chlorovirus* *Paramecium bursaria* chlorella virus 1 (PBCV-1) has a unique vertex where a spike structure is attached^15,16^; the capsids of mimiviruses Acanthamoeba polyphaga mimivirus (APMV) and Samba virus (SMBV) have stargate structures^17,18^. Both the unique vertex structure of PBCV-1 and the stargate structures of APMV and SMBV are thought to be relevant to the initiation of viral capsid assembly and host cell entry^16,18,19^. Such capsid components may be present in many other NCLDVs but have not been resolved due to technical challenges.

The large sizes of icosahedron-shaped NCLDVs (diameters: 150–500 nm) make it very challenging to attain high-resolution cryo-electron microscopy (cryo-EM) reconstructions of the viral capsids because of the Ewald sphere effect, thick vitreous ice needed for embedding the viral particles, and potential flexibility of the viral capsids^20–22^. Although numerous attempts have been made to determine the structures of NCLDV capsids^4–13,15–17^, only the capsids of PBCV-1 and African swine fever virus (ASFV) have been determined to high resolutions (PBCV-1: 3.5 Å; ASFV: 4.1 Å)^11–13^. However, icosahedral symmetries were imposed when calculating these two structures, which could have averaged out asymmetric capsid components. For PBCV-1, such components, though at low resolutions, have been observed in previous cryo-EM studies, and were averaged out in the 3.5-Å-resolution, icosahedrally averaged capsid structure^11,15,16^. Thus, detailed mechanisms on how asymmetric capsid components of NCLDV capsids are assembled remain unknown. In addition, because NCLDVs may start their assembly from their asymmetric capsid components (such as the unique vertex of PBCV-1 and stargate of APMV), insights on how capsid assembly of NCLDVs is initiated could be gained by resolving the asymmetric capsid components of these viruses at high resolutions.

PBCV-1 has an outer capsid with a diameter of ~190 nm, an inner bilayer lipid membrane that encloses a 331-kbp genome^23,24^, and a minor capsid shell in-between the outer capsid shell and the inner membrane^11^. Proteomic analyses of the PBCV-1 virion unexpectedly revealed 7 capsid-like proteins (A430L, A622L, A010R, A011L, A558L, A383R, and A384dL) in 5 different paralog classes, all with two highly conserved domains^24^. Our previously reported 3.5-Å-resolution, icosahedrally averaged, capsid structure of PBCV-1 shows a single type of pseudo-hexameric capsomer and pentameric capsomer in the outer capsid^11^. Previously reported low-resolution, fivefold averaged, cryo-EM structures of PBCV-1 showed that PBCV-1 has asymmetric capsid components^15,16^. However, to date, high-resolution structural information on these capsid components is unavailable.

Here we have extended the fivefold averaged structure of PBCV-1 capsid to near-atomic resolution (~3.8 Å) and obtained an atomic model of the viral capsid by employing a symmetry relaxation procedure to distinguish the unique vertex with the other capsid vertices of PBCV-1, and the “block-based” reconstruction method (see Methods)^22^. The atomic model contains 7135 polypeptide chains from 30 different kinds of proteins, with 15 kinds of the proteins previously unresolved. These previously unresolved proteins include five variants of the MCP that are involved in composing five types of pseudo-hexameric capsomers, one kind of penton protein variant that composes one type of pentameric capsomer, and nine kinds of minor capsid proteins. This is a near-atomic description of a NCLDV capsid that has resolved the asymmetric capsid components. Based on these observations, we propose a model for how asymmetric capsid components of PBCV-1 are assembled and how the capsid assembly is initiated, which may be similar in many other NCLDVs.

## Results

### Overall fivefold averaged structure of PBCV-1

**Fig. 1:**
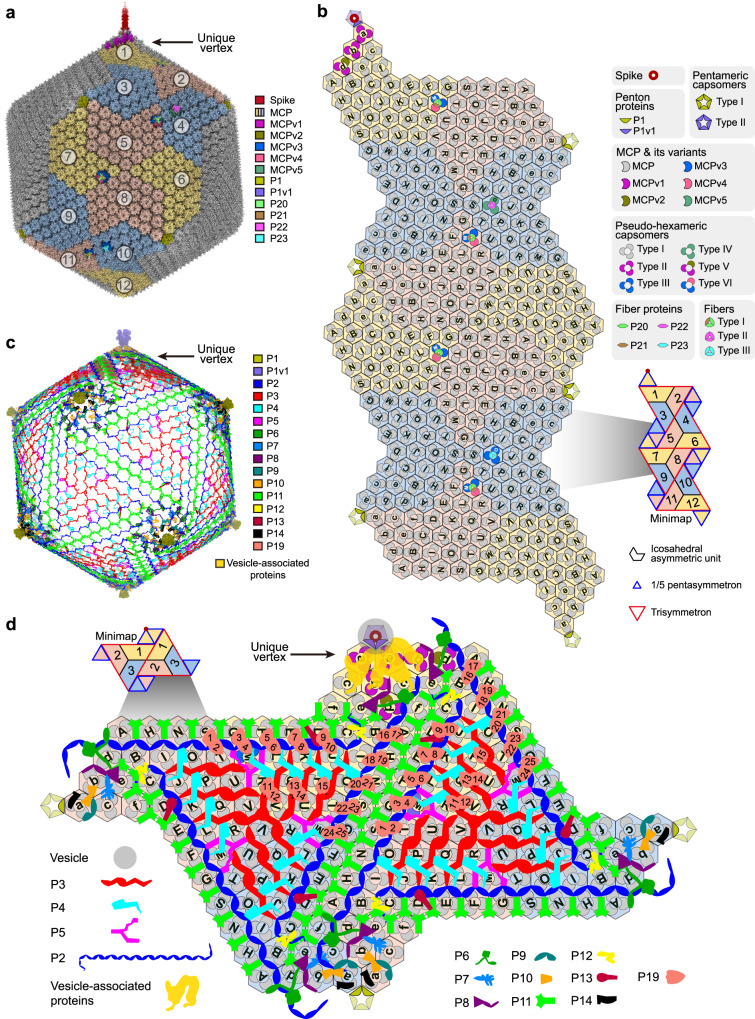
Overall structure of the PBCV-1 capsid. **a** Fivefold averaged cryo-EM map of the viral capsid. Cryo-EM densities of the spike and proteins within one fivefold asymmetric unit are colored according to the color key. The MCPs within neighboring icosahedral asymmetric units are in alternate colors. The icosahedral asymmetric units within the fivefold asymmetric unit are labeled 1, 2, 3, … The unique vertex is indicated with a black arrow. **b** Diagrammatic organization of the outer capsid, viewed from outside the virus. The pseudo-hexameric capsomers are outlined using hexagons, and labeled A, B, C, … in the trisymmetrons and a, b, c, … in the pentasymmetrons^11^. Lighter colors are used for proteins from neighboring fivefold asymmetric units. **c** Cryo-EM densities of the minor capsid proteins that bind the inner surface of the outer capsid, the penton protein and penton protein variant. The unique vertex is indicated with a black arrow. **d** Diagrammatic organization of the minor capsid proteins and capsomers around the unique vertex, viewed from inside the virus. The pseudo-hexameric and pentameric capsomers and the spike are shown as in **b**. The vesicle-associated proteins are minor capsid proteins P9, P15, P16, P17, and P18.

By means of a symmetry relaxation procedure and the “block-based” reconstruction method^22^ (see Methods), we distinguished between the unique vertex topped by a spike structure and the other eleven vertices (hereafter “common vertex”) of the PBCV-1 capsid, obtaining a ~3.8-Å-resolution cryo-EM structure imposing fivefold (C5) symmetry (Fig. 1a–c and Supplementary Figs. 1 and 2). Each fivefold asymmetric unit contains 12 icosahedral asymmetric units (numbered from 1 to 12) (Fig. 1a). This cryo-EM map allows us to model the PBCV-1 capsid at near-atomic resolution, leading to the identification of ten kinds of previously unresolved minor capsid proteins (P15, P16, P17, P18, P19, P20, P21, P22, P23, and the penton protein variant P1v1) and five kinds of MCP variants (MCPv1, MCPv2, MCPv3, MCPv4, and MCPv5) (Supplementary Tables 1 and 2).

The outer capsid shell of the PBCV-1 structure consists of the MCP (Vp54), five MCP variants, penton protein P1, and penton protein variant (P1v1) (Fig. 1a–d and Supplementary Movie 1). The MCP and its variants are assembled as pseudo-hexameric trimers, while the penton protein and its variant are assembled as pentamers (Fig. 1b). The pseudo-hexameric capsomers form 20 trisymmetrons and 12 pentasymmetrons (Fig. 1b and Supplementary Fig. 3a). In addition, the center of each pentasymmetron is filled by a pentameric capsomer (Supplementary Fig. 3a).

Each fivefold asymmetric unit of the viral capsid contains twelve icosahedral asymmetric units (Fig. 1a, b and Supplementary Movie 1), with each icosahedral asymmetric unit containing 28 pseudo-hexameric capsomers (22 of these in the trisymmetron: capsomer A, B, C…; six of these in the pentasymmetron: capsomer a, b, c, …) (Fig. 1b). Although the majority of the pseudo-hexameric capsomers within each fivefold asymmetric unit are entirely composed of the MCP, nine out of the 336 pseudo-hexameric capsomers contain MCP variants (Fig. 1b). Similarly, the composition of the pentameric capsomer in the unique vertex is different than that in the other eleven common vertices (unique vertex: P1v1; common vertex: P1). Minor capsid proteins P2, P3… P19 form a network below the outer capsid shell (Fig. 1c and Supplementary Movie 2). P2, P3, P4, P5, P6, P8, P9, P11, P12, and P13 are universally present in all the twelve icosahedral asymmetric units, whereas P15, P16, P17, P18, P19, and P7, P10, P14 are only present in icosahedral asymmetric unit 1 (at the unique vertex) and the other eleven icosahedral asymmetric units, respectively (Fig. 1d and Supplementary Table 1). Besides the minor capsid proteins below the outer capsid shell, minor capsid proteins P20, P21, P22, and P23 were found to form fiber structures that are attached to capsomer M in icosahedral asymmetric unit 1, 5, 7, and 11, and capsomer O in icosahedral asymmetric unit 4 and 10 (Fig. 1a, b and Supplementary Movie 1).

### Variants of the major capsid protein and penton protein

**Fig. 2:**
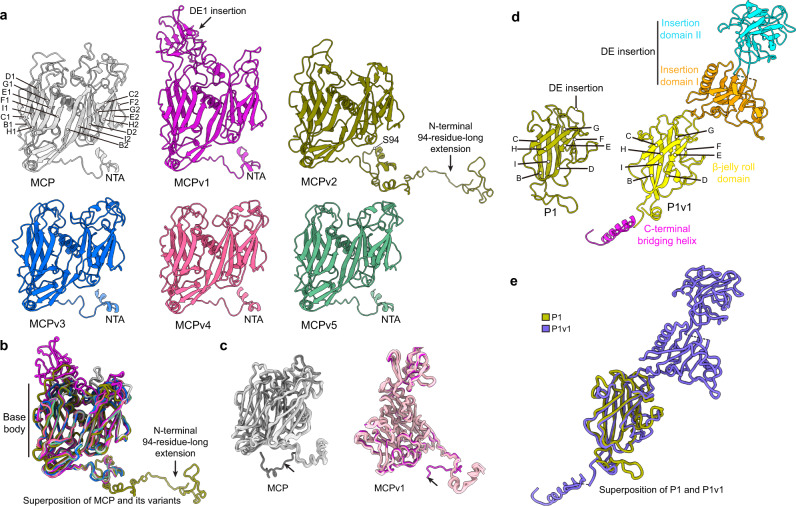
Structures of the MCP, MCP variants, penton protein, and penton protein variant. **a** Ribbon diagrams of the MCP and its variants. **b** Backbone superposition of MCP and its variants (MCPv1, MCPv2, MCPv3, MCPv4, and MCPv5), colored as in **a**. **c** Backbone superpositions of all the MCPs (left) and all the MCPv1s (right) in the pentasymmetrons of the unique vertex. Black arrows indicate the conformational rearrangements in the NTAs of a MCP subunit of capsomer b and a MCPv1 subunit of capsomer a in icosahedral asymmetric unit 1. **d** Ribbon diagrams of the penton protein P1 and its variant P1v1. **e** Backbone superposition of P1 (olive green) and P1v1 (violet blue).

Like the MCP, all five MCP variants consist of an N-terminal arm (NTA) and two sequential “jelly-roll” folds^11,25^ (Fig. 2a), with each jelly roll consisting of eight anti-parallel β-strands (B, C, D, E, F, G, H, I), four exterior inter-strand insertions (BC, DE, FG, and HI insertion) and three interior inter-strand insertions (CD, EF, and GH insertion). The exterior inter-strand insertions form protrusions on the outer surface of the capsomers, while the β-strands and interior inter-strand insertions of the jelly rolls (hereafter “base body”) form the base regions of the capsomers (Supplementary Fig. 4). In addition, MCPv2 has an additional N-terminal 94-residue-long extension, compared with the MCP and the other MCP variants (Fig. 2a, b). Sequence alignment showed that the sequence identities between the MCP and its variants range from 25.6 to 42.8% (Supplementary Fig. 4a and Supplementary Table 3). However, sequence identities between their base bodies (ranging from 40.9% to 58.2%) are significantly higher than those of the exterior inter-strand insertion regions (ranging from 4.0% to 22.2%). Moreover, the structures of the base bodies of the MCP and its variants can be well aligned with each other (Fig. 2b), with occasional conformational rearrangements of the N-terminal arm region in a MCPv1 subunit of capsomer b and a MCP subunit of capsomer c in icosahedral asymmetric unit 1 (Fig. 2c), probably caused by associations with the minor capsid proteins P17 and P18 (see below). On the other hand, the structures of the exterior inter-strand insertion regions are highly variable in insertion lengths and glycosylation sites (Fig. 2b, Supplementary Fig. 4a, and Supplementary Table 4). MCPv1 has a very large DE1 insertion (residues 77–188), forming an ~5-nm-high protrusion on the exterior of the capsomer (Fig. 2a and Supplementary Fig. 4). The HI1 and DE2 insertions of MCPv2, MCPv3, MCPv4 and MCPv5 are significantly shorter (Supplementary Fig. 4a), when compared with those of the MCP. Several glycosylation sites were observed in the exterior insertions of the MCP and its variants (Supplementary Fig. 4a and Supplementary Table 4), with all the glycosylation sites associated with a sequon system that is likely unique to chloroviruses^11,25^. Mainly because of the sequence variations in the exterior insertion regions, the numbers and positions of the glycosylation sites among the MCP and its variants also have very high variations (Supplementary Fig. 4a and Supplementary Table 4).

Both the penton protein P1 and its variant P1v1 consist of a single jelly-roll fold composed of eight anti-parallel β-strands (B, C, D, E, F, G, H, I) and inter-strand insertions^11^, but P1v1 has an additional C-terminal bridging helix (residues 491–524) (Fig. 2d, e). The jelly-roll regions of P1 and P1v1 show nearly identical structures (Fig. 2e), although P1 and P1v1 share low sequence identity (~10%) (Supplementary Fig. 5 and Supplementary Table 3). However, P1v1 has a much larger DE insertion than P1, which folds into two additional β-strand enriched domains, namely insertion domain I (residues 59–203 and 370–385) and II (residues 210–367) (Fig. 2d, e and Supplementary Fig. 5a). In addition, the potential numbers and sites of glycosylation in their exterior insertions are significantly different from each other (Supplementary Fig. 5a and Supplementary Table 4).

### Pseudo-hexameric and pentameric capsomers

**Fig. 3:**
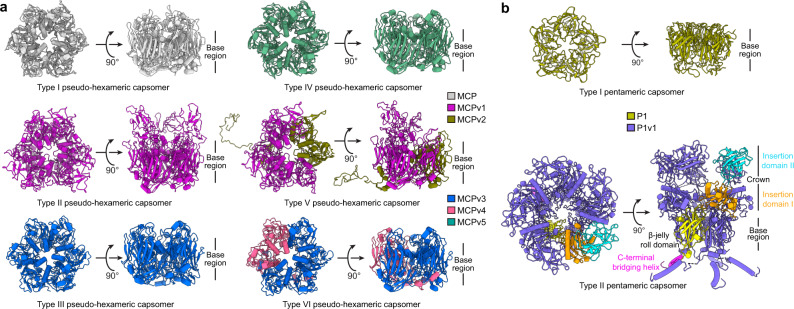
Structures of the pseudo-hexameric and pentameric capsomers. **a** Ribbon diagrams of all six types of pseudo-hexameric capsomers. **b** Ribbon diagrams of the two types of pentameric capsomers. The C-terminal bridging helix, β-jelly roll domain, insertion domain I and insertion domain II of one P1v1 molecule in the type II pentameric capsomer are colored magenta, yellow, orange, and cyan, respectively.

Each pseudo-hexameric capsomer is composed of three monomers of the MCP and/or its variants, resulting in six types of capsomers, namely, type I, II, III, IV, V, and VI (Fig. 3a). Type I, type II, type III and type IV capsomers are homotrimers of the MCP, MCPv1, MCPv3 and MCPv5, respectively, while type V and type VI capsomers are heterotrimers, with type V capsomer composed of two MCPv1 and one MCPv2 subunits, and type VI capsomer composed of two MCPv3 and one MCPv4 subunits (Figs. 1b and 3a). Different types of the pseudo-hexameric capsomers do not distribute uniformly among the twelve icosahedral asymmetric units within each fivefold asymmetric unit of the PBCV-1 capsid (Fig. 1a, b). Type I capsomers are present in all the icosahedral asymmetric units, while type II and type V capsomers are only present in the unique vertex, with type II capsomers located at position a, b and type V capsomer located at position d, respectively. Type III and type IV capsomers are located at position O of icosahedral asymmetric unit 10 and 4, respectively. Type VI capsomers are located at position M of icosahedral asymmetric unit 1, 5, 7, and 11.

Like the monomer structures of the MCP and MCP variants, all these six types of pseudo-hexameric capsomers have highly varied structures in the exterior inter-strand insertion regions of the jelly-roll folds, but nearly identical backbone structures in the base region (Fig. 3a and Supplementary Fig. 6a) presumably for maintaining the overall icosahedral symmetry of the viral capsid. Although the backbone structures in these regions are highly conserved, numerous amino-acid substitutions are observed on the surfaces of type II, III, IV, V and VI capsomers when compared to that of type I capsomer (Supplementary Fig. 6b). Such structural variations in the exterior insertion and base regions could determine the binding specificity between different types of pseudo-hexameric capsomers and their adjacent capsid components.

Each pentameric capsomer in the eleven common vertices of the viral capsid (hereafter “type I pentameric capsomer”) is composed of five penton protein (P1) monomers, whereas the pentameric capsomer in the unique vertex (hereafter “type II pentameric capsomer”) is composed of five monomers of the penton protein variant (P1v1) (Fig. 3b). Compared with the type I pentameric capsomer, the type II pentameric capsomer has an ~7-nm-high “crown” structure formed by the DE insertion of its five P1v1 subunits (Fig. 3b). Besides, the type II pentameric capsomer has additional C-terminal bridging helices that extend to adjacent pseudo-hexameric capsomers. However, like the pseudo-hexameric capsomers, the backbone structures in the base regions of the two types of pentameric capsomers are nearly identical, yet with amino-acid substitutions observed on almost the entire surface of the type II pentameric capsomer, when compared to type I pentameric capsomer (Supplementary Fig. 6c, d).

### Pentasymmetron in the unique vertex

The structures of the pentasymmetrons located in the eleven common vertices of the viral capsid show no significant differences from what was previously described in the icosahedrally averaged capsid structure of PBCV-1^11^, with each of these pentasymmetrons containing 30 type I pseudo-hexameric capsomer and one type I pentameric capsomer (Fig. 1b). All capsomers within each pentasymmetron in the common vertices are crosslinked together by minor capsid proteins P6, P7, P8, P9, P10 and P14. By contrast, the pentasymmetron in the unique vertex of the viral capsid contains up to three types (type I, II and V) of pseudo-hexameric capsomers and a type II pentameric capsomer (Fig. 1a, b). Furthermore, minor capsid proteins P7, P10 and P14 are missing, while P15, P16, P17 and P18 are observed in this pentasymmetron (Fig. 1c, d and Supplementary Table 1).

On top of the type II pentameric capsomer in the unique vertex is a spike structure (Fig. 1a)^15,16^, whose cryo-EM density is uninterpretable probably due to a symmetry mismatch with the viral capsid. The spike structure attaches to the capsomer mainly by inserting its base into a channel created by the insertion domain I of the penton protein variant P1v1 (Supplementary Fig. 7). The association between the type II pentameric capsomer and the spike is reinforced by the insertion domain II of P1v1 (Supplementary Fig. 7).

**Fig. 4:**
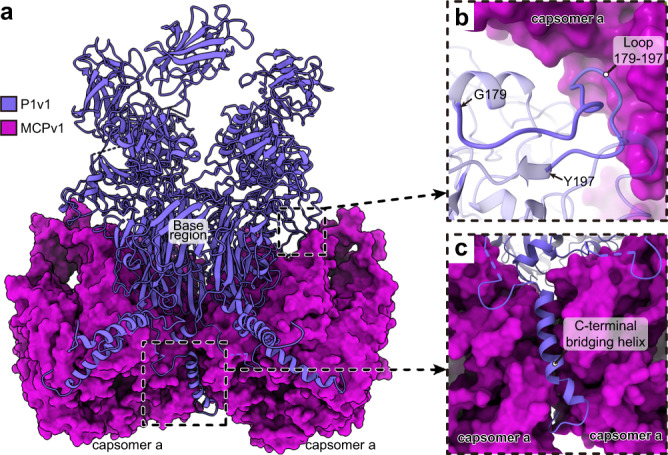
Associations between the type II pentameric capsomer and neighboring pseudo-hexameric capsomer a in the unique vertex. **a** Structures of the type II pentameric capsomer (cornflower blue) and its two adjacent pseudo-hexameric capsomers (magenta). **b**, **c** Zoomed-in views of the boxed regions in **a**, showing the contacts between the loop 179–197 (**b**), C-terminal bridging helix (**c**) of P1v1, and capsomer a.

Like the type I pentameric capsomers in the common vertices, the type II pentameric capsomer in the unique vertex makes contacts with its neighboring capsomers (capsomer a) mainly through the base region. However, the association between the type II pentameric capsomer and its neighboring capsomers is reinforced by loop 179–197 of insertion domain I and the C-terminal bridging helix of P1v1 (Fig. 4a–c). The loop 179–197 binds to the top of capsomer a (Fig. 4b), while the C-terminal bridging helix cements the gap between two neighboring capsomer a’s (Fig. 4c). In this way, the type II pentameric capsomer is tightly crosslinked to capsomer a in the unique vertex.

**Fig. 5:**
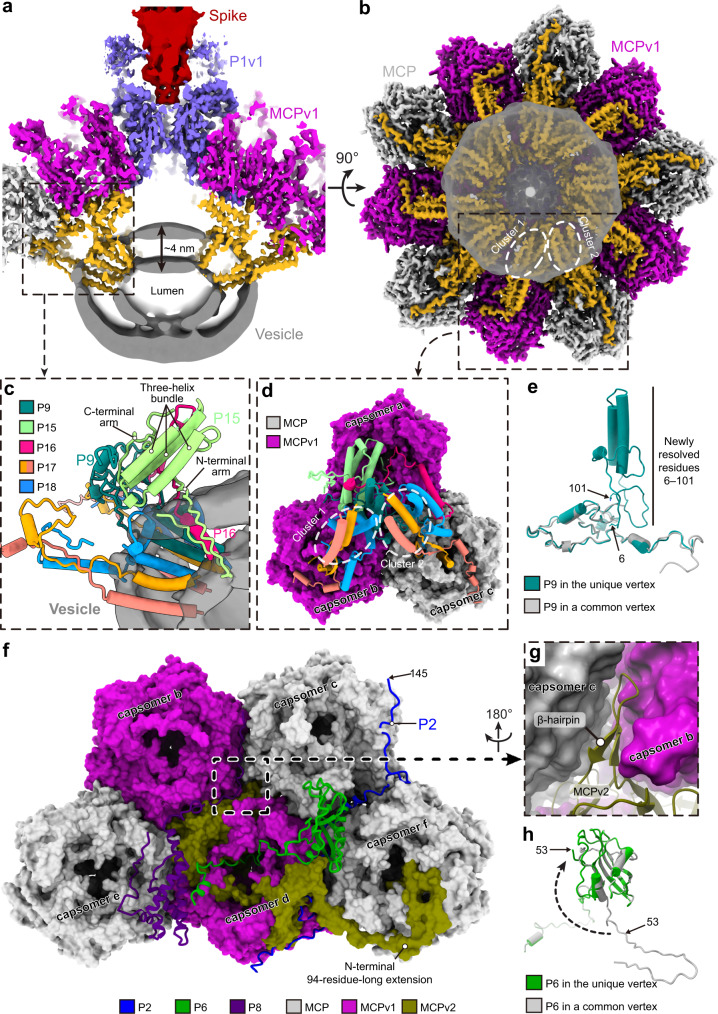
Associations between minor capsid proteins P2, P6, P8, vesicle-associated proteins and pseudo-hexameric capsomers, and between neighboring capsomers in the pentasymmetron of the unique vertex. **a** A slab view of the cryo-EM density around the type II pentameric capsomer in the unique vertex. The ~4-nm-thick lipid bilayer of the vesicle (dark gray) is associated with the capsid via the vesicle-associated proteins (golden). **b** The cryo-EM density around the vesicle region viewed from inside the viral capsid. The two transmembrane helix clusters formed by the vesicle-associated proteins are highlighted using dashed ovals. **c** Zoomed-in view of the boxed region in **a**, showing the structures of vesicle-associated proteins within one fivefold asymmetric unit. **d** Zoomed-in view of the boxed region in **b**. The pseudo-hexameric capsomers are labeled as in Fig. 1b. **e** Superposition of the structures of minor capsid protein P9 in the unique vertex and a common vertex. **f** Structures of P2, P6 and P8, and their neighboring capsomers. The minor capsid proteins and capsomers are shown as ribbon diagrams and surfaces, respectively. **g** Zoomed-in view of the boxed region in **f**, showing the contacts between the β-hairpin of MCPv2 and capsomers b and c. **h** Superposition of P6 in the unique vertex and a common vertex. The dashed curved arrow indicates the conformational rearrangement of the N-terminal arms of P6. All the pseudo-hexameric capsomers are labeled as in Fig. 1b.

Below the type II pentameric capsomer is a lipid vesicle that has an ~7.3 × 10^4^-Å^3^-large lumen, which is supported by minor capsid proteins P9, P15, P16, P17, and P18 (Fig. 5a–c and Supplementary Movie 2). P15 binds to the bottom of capsomer a via its three-helix bundle and C-terminal arm (Fig. 5c, d), and inserts its N-terminal arm into the bilayer membrane of the vesicle structure below the type II pentameric capsomer (Fig. 5c). In addition, P15 makes extensive contacts with P9 and P16 (Fig. 5d). Like in the pentasymmetron of the common vertices, P9 in the unique vertex binds to the gaps between capsomer a and b, and between capsomer a and c, holding these capsomers together^11^. However, in the unique vertex, capsomer a and b are no longer type I pseudo-hexameric capsomers but replaced by the type II pseudo-hexameric capsomers (Figs. 1b and 5d). In addition, residues 6–101 of P9 are resolved (Fig. 5e), probably due to the support from P15 and P16 (Fig. 5c, d). The resolved N-terminal region of P9, consistent with its previously predicted role in membrane association^11^, forms two transmembrane helices (residues 34–55 and 61–78) that are inserted in the bilayer membrane of the vesicle structure (Fig. 5c). Surrounding the transmembrane region of P9 is a transmembrane helix (residues 9–25) of P16 and two small clusters of membrane-associated helices, with each cluster composed of six helices contributed by two copies of P17 and one copy of P18 (Fig. 5d). P17 and P18 of each cluster, besides their association with the vesicle structure, extend to the bottoms of capsomer b and c and make contacts with them (Fig. 5d). In this way, capsomers a, b, and c are glued together, and the vesicle structure is firmly associated with the unique vertex.

Capsomer d in the unique vertex is a hetero-trimer composed of two MCPv1 monomers and one MCPv2 monomer. Unlike other types of pseudo-hexameric capsomers, it uses a β-hairpin structure in the BC2 insertion and an N-terminal 94-residue-long extension of the MCPv2 monomer to reinforce the association with its neighboring capsomers (Fig. 5f). The β-hairpin structure is inserted in the gap between the outer surfaces of capsomer b and c (Fig. 5f, g). The N-terminal 94-residue-long extension of the MCPv2 monomer forms a fiber-like structure, running along the gap between capsomer d and capsomer A in the neighboring trisymmetron, finally stopping at the central base of capsomer f (Fig. 5f).

Like in the common vertices, capsomers e and f in the unique vertex are crosslinked to their capsomer neighbors by minor capsid proteins P6, P8, and the tape-measure protein P2 (Figs. 1d and 5f). However, the N-terminal arm of P6 in the unique vertex moves away from the gap between capsomer d and capsomer A in the neighboring trisymmetron (Fig. 5h). Instead, this gap is filled by the N-terminal 94-residue-long extension of MCPv2 in the unique vertex as mentioned above (Figs. 1d and 5f). In addition, tape-measure protein P2, which makes contacts with all the pseudo-hexameric capsomers within each pentasymmetron in the common vertices^11^, only makes contacts with capsomer c, d, e, and f (but not a or b) in the unique vertex (Fig. 1d). Residues 94–144 of P2, which interact with capsomers a and b in the common vertices, are disordered in the unique vertex (Fig. 5f). The C terminus of the tape-measure protein then extends from the pentasymmetron in the unique vertex to its neighboring trisymmetron.

### Trisymmetron in the unique vertex

**Fig. 6:**
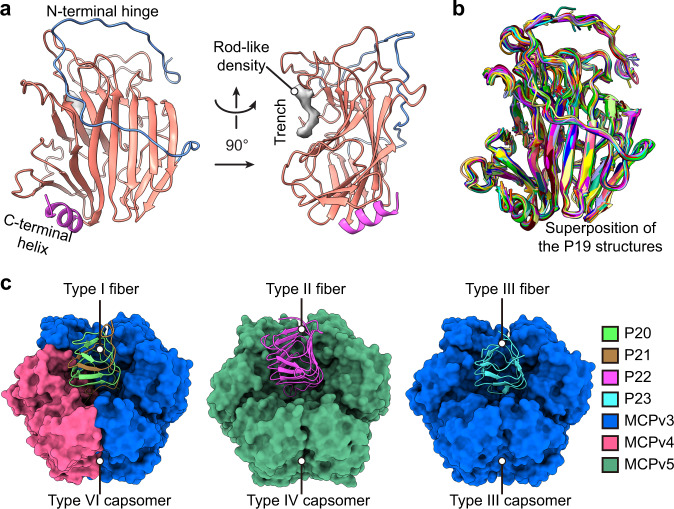
Structures of the finger proteins and fibers. **a** Ribbon diagram of one copy of the finger protein, P19, with the rod-like cryo-EM density in its trench. **b** Superposition of the 25 copies of the P19 proteins within one fivefold asymmetric unit. **c** Structures of the three types of fibers (shown as ribbon diagrams) and their associated capsomers (shown as surfaces).

The construction of the trisymmetron in the unique vertex is contributed by capsomers A to V within icosahedral asymmetric unit 1, 2, and 3, and minor capsid proteins below the capsomers (Fig. 1b). The major difference between the trisymmetron in the unique vertex and those in the common vertices is the presence of minor capsid protein P19 (or “finger protein”^15,16^) below the capsomers within the trisymmetron in the unique vertex (Fig. 1d and Supplementary Movie 2). Twenty-five copies of finger proteins are observed and modeled within each fivefold asymmetric unit (Figs. 1d and 6a). The cryo-EM densities of each finger protein conformer become weaker the further they are from the unique vertex, indicating likely varied occupancies among these finger protein conformers (Supplementary Fig. 8). The modeled structures of the finger protein conformers are nearly identical (Fig. 6b), with each consisting of an N-terminal hinge (residues 48–93), an arch-like anti-parallel β-sandwich (residues 94–328) and a C-terminal helix (residues 329–339) (Fig. 6a). A rod-like electron density was observed in the trench of the β-sandwich region of each finger protein conformer, which might be contributed by a ligand (Fig. 6a). Dali search^26^ results showed that the structure of the finger protein is close to the structures of some polysaccharide lyase families or their sub-domains (such as ɑ-mannosidase, β-galactosidase, and alginate lyase). In addition, the polysaccharide lyase vAL-1 of chlorovirus CVK2 also show similar β-sandwich folds^27^. Therefore, the finger protein may also function as a polysaccharide lyase that is used to help degrade the host cell wall during infection.

### Fiber association

Previous cryo-EM studies showed that PBCV-1 has multiple fiber attachment sites at the outer surface of the capsid^15,16^. We found six fiber-attached capsomers out of the 336 capsomers within each fivefold asymmetric unit of the current cryo-EM map (type VI pseudo-hexameric capsomer M in icosahedral asymmetric unit 1, 5, 7, and 11; type III and IV pseudo-hexameric capsomer O in icosahedral asymmetric unit 10 and 4, respectively) (Fig. 1a, b and Supplementary Movie 1). Probably because of flexibility, only a short segment (~4-nm long), which is proximal to the capsomer surfaces, of each fiber is resolved in the cryo-EM map^15,16^. All the fiber structures have a right-handed parallel β-helix trimer structure, resembling the β-helix structure of gp34 of bacteriophage T4^28^.

There are three types of fiber structures constructed by minor capsid proteins P20, P21, P22, and P23. The type I fiber structure is composed of a heterotrimer of two P20 subunits and one P21 subunit, while the type II and type III fiber structures are composed of homotrimers of P22 and P23, respectively (Fig. 6c). The type I fiber has four structurally nearly identical conformers, with each of them attached to a capsomer M (type VI pseudo-hexameric capsomer) in icosahedral asymmetric unit 1, 5, 7, and 11 (Figs. 1a, b and 6c and Supplementary Movie 1). The capsomer O in icosahedral asymmetric unit 4 (type IV pseudo-hexameric capsomer) and 10 (type III pseudo-hexameric capsomer) are connected to type II and type III fibers, respectively (Fig. 6c). The backbone structures of the type VI, type IV and type III pseudo-hexameric capsomers are nearly identical (Supplementary Fig. 6e). However, the residues located in the outer surfaces of these three types of capsomers are significantly different from each other, which could be used for selectively recruiting the three types of fibers (Supplementary Fig. 6f).

## Discussion

About four times the data used for the previously reported, icosahedrally averaged reconstruction of PBCV-1^11^ (~56,500 particles) were used to generate the present fivefold averaged reconstruction of PBCV-1. In theory, to achieve the same resolution as that of the icosahedrally averaged reconstruction (3.5 Å), this fivefold averaged reconstruction requires twelve times larger data than what was used for the previously reported icosahedrally, averaged reconstruction. This could explain why the resolution of the present fivefold averaged reconstruction of PBCV-1 is slightly worse than the previously achieved 3.5 Å for the icosahedrally averaged reconstruction of PBCV-1^11^.

Unlike other icosahedral viral capsids with available atomic models (to our knowledge), the fivefold averaged PBCV-1 capsid structure described here shows up to six kinds of MCPs and two kinds of penton proteins, resulting in six types of pseudo-hexameric and two types of pentameric capsomers. Although the sequence identities between the MCP and its variants and between the penton protein and its variant are not high (Supplementary Figs. 4 and 5 and Supplementary Table 3), the mainchain structures of the portions of the MCP (and its variants) and the penton protein (and its variant) that form the base region of the capsomers remain nearly unchanged between the MCP and its variants and between the penton protein and its variant (Fig. 2b–e). As a result, the mainchain structures of the base regions are nearly identical among different types of pseudo-hexameric capsomers and among different types of pentameric capsomers. This may be required for maintaining the structural integrity of the outer capsid shell of the virus. On the other hand, the surfaces among different types of pseudo-hexameric capsomers and among different types of pentameric capsomers vary significantly (Supplementary Fig. 6b, d), creating local capsid microenvironments that do not follow the overall icosahedral symmetry of the viral capsid. These microenvironments are used to associate with auxiliary capsid components that do not follow the overall icosahedral symmetry of the viral capsid but play critical roles in the life cycle of PBCV-1^16,19^. For example, the spike structure is associated with the type II pentameric capsomer (Supplementary Fig. 7). Type I, type II, and type III fibers are attached to type VI, type IV and type III pseudo-hexameric capsomers, respectively (Fig. 6c); minor capsid proteins P15, P16, P17, P18 and P19 are only present in the unique vertex of the PBCV-1 capsid where type II and type V pseudo-hexameric capsomers and type II pentameric capsomer appear (Fig. 1d). Fiber protein P22 (aka, A122/123R; aka, Vp260)^16,29^ is one of the three PBCV-1 hypermutable virion-associated proteins observed in an experimental coevolution experiment with the *Chlorella* host, where the 14 observed mutations are highly repeatable, non-synonymous and forward selected, inferring an essential role in virus life cycle completion^30^. Blast searches identified multiple Vp54 (the MCP) homologs from Phycodnaviruses such as *Paramecium bursaria* Chlorella virus NY-2A, *Paramecium bursaria* Chlorella virus KS-1B and Phaeocystis globosa virus, and Mimiviruses such as APMV and Cafeteria roenbergensis virus (CroV), and an Abalone asfa-like virus that is phylogenetically close to Asfarviruses, suggesting the possible presence of multiple kinds of pseudo-hexameric capsomers in its viral capsid. In a previously constructed phylogenetic tree of NCLDVs^31^, both APMV and CroV were assigned to Mimiviruses clade 18 that is sister to Late Phycodnaviruses where PBCV-1 was assigned, whereas Asfarviruses are more phylogenetically distant to PBCV-1. Thus, similar mechanisms may be used by many other NCLDVs to associate with auxiliary capsid components that does not follow the overall icosahedral symmetry of their capsids to fulfill critical functions in their life cycles.

The assembly of the PBCV-1 capsid appears to be initiated from the type II pentameric capsomer in the unique vertex. The type II pentameric capsomer recruits the spike structure and five capsomer a’s (type II pseudo-hexameric capsomers). The type II pentameric capsomer and the five type II pseudo-hexameric capsomers are further crosslinked by the loop 179–197 of insertion domain I and C-terminal bridging helix of P1v1. Capsomer b (type II pseudo-hexameric capsomer), c (type I pseudo-hexameric capsomer), as well as P15, P16, P17 and P18 are recruited and form a vesicle structure below the type II pentameric capsomer. Capsomer d (type V pseudo-hexameric capsomer) binds to capsomer b (type II pseudo-hexameric capsomer) and c (type I pseudo-hexameric capsomer), and uses a β-hairpin structure of its MCPv2 subunit to reinforce the association with capsomer b and c. Capsomer e and f are crosslinked to its neighboring capsomers in the pentasymmetron by P6, P8 and the N-terminal 94-residue-long extension of the MCPv2 subunit of capsomer d. The C terminus of the tape measure protein then extends from the pentasymmetron in the unique vertex to its neighboring trisymmetron to continue the viral capsid assembly. A similar mechanism may be used by many other NCLDVs to initiate their capsid assembly. Initiating capsid assembly from a unique vertex seems to be a general mechanism that is also used by many other double-stranded DNA viruses, including tailed bacteriophages such as T4 and P22^32–35^, and herpesviruses^36^ for examples. Like PBCV-1, these viruses generally have large genomes that are capable of coding proteins specially associated with their unique vertex which is used not only to initiate capsid assembly but also to fulfill other crucial viral functions such as genome encapsidation and/or genome exit^36^.

## Methods

### PBCV-1 virion preparation

Virus PBCV-1 was grown by infection of *Chlorella variabilis* NC64A cells as described previously^37^. The virus purification procedure is described elsewhere^38^. Briefly, 3-day old chlorella cell lysates were treated with Triton X-100 (Fisher Chemical) (to 1%) at room temperature with constant mixing for 1 h. Then cell lysates were centrifuged at 4000 × *g* for 5 min at 4 °C. The supernatant was then centrifuged at 4 °C for 50 min at 53,000 × *g* to pellet the virus. The virus pellet was resuspended in a small volume of virus suspension buffer (VSB, 50 mM Tris-HCl, 10 mM MgCl_2_ pH 7.8) and placed on 10–40% linear sucrose density gradients equilibrated with VSB in a swinging bucket rotor and centrifuged at 4 °C for 20 min at 72,000 × *g*. The virus band was removed from the gradient with a sterile needle, diluted with VSB, and centrifuged for 50 min at 53,000 × *g* to pellet the virus. The virus in the pellet was re-suspended and treated with proteinase K (0.02 mg/ml) (Promega) for 1 h at 45 °C. After proteinase K treatment the virus was subjected to a second sucrose density gradient centrifugation and resuspension.

### Genome resequencing

The updated cryo-EM structure (this paper) revealed sequence discrepancies in the PBCV-1 genome. To identify potential erroneous regions of DNA, we re-sequenced regions of the virus genome that did not fit the cryo-EM structure. Virus DNA was amplified by PCR using several custom primer sets (Supplementary Table 5) and confirmed on 1% agarose gels. Successfully amplified products were verified by bidirectional Sanger sequencing (Genewiz, South Plainfield, NJ). The two following PBCV-1 genes were re-examined: *a533r*—to re-sequence a PBCV-1 penton protein because it was noticeably shorter than its chlorovirus homologs (236 nucleotides were missing in the genome), and *a383r*—to re-sequence a PBCV-1 capsid protein because it did not fit the cryo-EM model and after closer inspection it was also shorter than its chlorovirus homologs and interestingly, exhibited an ~500 nucleotide gap between the next gene.

### Cryo-EM data collection and processing

About 3 μl aliquots of the PBCV-1 sample were frozen onto glow-discharged Lacey carbon EM grids (Ted Pella) using a CP3 freezer (Gatan) with a blot time of 6 s. The cryo-EM data were collected using an FEI Titan Krios EM (ThermoFisher Scientific) operated at 300 kV. All cryo-EM images were recorded on a K2 Summit detector (Gatan). We used an exposure time of ~8 s, dose fractionated into 40 frames. A total of ~16,200 movie stacks were automatically collected using the Leginon program with a dose rate of ~8 e^−^/(pixel·s) and a nominal magnification of ×18,000 in either the super-resolution or counting mode (corresponding to a physical pixel size of 1.62 Å). The movie stacks were subjected to motion correction and dose weighting using the MotionCorr2 program^39^. The contrast transfer function (CTF) parameters of each micrograph were estimated using the program CTFFIND4^40^. The viral particles were automatically picked using Appion^41^ or Gautomatch (developed by Kai Zhang, https://www2.mrc-lmb.cam.ac.uk/download/gautomatch-056/) and sorted by two-dimensional (2D) classification using the RELION program^42^, resulting in a total of ~56,500 particles for further data processing (Supplementary Table 6).

For three-dimensional (3D) refinement, we used a previously reported cryo-EM reconstruction of PBCV-1^16^, low pass filtered to 40 Å resolution, as the initial model, and determined the alignment parameters of each particle using the jspr program^43^, assuming icosahedral symmetry. This gave us an icosahedrally averaged, 3D reconstruction of the PBCV-1 capsid. The symmetry of the reconstruction was then relaxed to C5 using a previously reported procedure^16^, resulting in a fivefold averaged, 3D reconstruction of PBCV-1. This fivefold averaged reconstruction was then further improved by employing a block-based reconstruction method to account for defocus gradient and potential flexibility of the viral particles^22^, giving us the final fivefold averaged, reconstruction of PBCV-1 at ~3.8 Å resolution. The resolution estimations were performed based on the gold-standard Fourier shell correlation (FSC) = 0.143 criterion^44^ (Supplementary Table 6).

### Model building and refinement

The fivefold averaged cryo-EM map of PBCV-1 was initially interpreted by fitting the previously reported atomic model of the icosahedrally averaged, cryo-EM reconstruction of PBCV-1 into the cryo-EM densities as a rigid body. The polypeptide chains that did not have corresponding densities in the cryo-EM map (e.g., minor capsid proteins around the unique vertex) were removed from the model. Each polypeptide chain within the resultant model was then inspected in Coot^45^. The polypeptide chains of the MCP variants and penton protein variant were identified by their loop lengths, sidechain size distributions, and/or glycosylation site distributions and remodeled in Coot^45^. Because the cryo-EM densities of the insertion domain II of the penton protein variant P1v1 were poor, probably due to flexibility, a predicted model (computed using RoseTTAFold^46^) was fitted into the cryo-EM density for the insertion domain II of P1v1. The cryo-EM densities of the other polypeptide chains, except for the ones that have less than 30 residues or do not have prominent side-chain information (Supplementary Fig. 3b), were interpreted by manually building the Cα models in Coot^45^. To assign sequences to these Cα models, we compared them with in silico structures (computed using AlphaFold2^47^ or RoseTTAFold^46^) of all 148 proteins that had been previously detected in the PBCV-1 virion in a proteomic study (Supplementary Fig. 9)^24^. For the Cα models of minor capsid proteins P16, P18, and P19, a single in silico structure was immediately matched with each of them (Supplementary Fig. 9). For the Cα models of minor capsid proteins P15, P17, P20, P21, P22, and P23, a single sequence was unambiguously selected for each of them by using additional information on side-chain size and/or glycosylation site distributions observed in the cryo-EM densities (Supplementary Fig. 9). The sequence assignments were further validated using local inter-molecular chemical environments such as hydrophobic and potential charge interactions. The transmembrane helix regions in the resulting atomic models of minor capsid protein P16, P17, and P18 were supported by performing transmembrane helix predictions using TMHMM 2.0^48,49^, HMMTOP 2.0^50,51^, and Phobius^52,53^. Because the cryo-EM densities of the glycans are poor, they were not modeled. The atomic model of the unique vertex and previously unresolved polypeptide chains in the other regions of the fivefold averaged, cryo-EM map of PBCV-1 was then refined using the Phenix^54^ and Rosetta program^55^ (Supplementary Table 6). Molecular graphics were generated using Chimera^56^ and ChimeraX^57^.

### Reporting summary

Further information on research design is available in the Nature Research Reporting Summary linked to this article.

## Supplementary information

Supplementary Information

Description of Additional Supplementary Files

Supplementary Movie 1

Supplementary Movie 2

Reporting Summary

## Data Availability

The fivefold averaged cryo-EM reconstruction before and after applying the “block-based” reconstruction method of PBCV-1 have been deposited in the Electron Microscopy Data Bank (EMDB) under the accession numbers EMD-34439 and EMD-34438, respectively. The atomic model of the unique vertex and previously unresolved polypeptide chains in the other regions of the PBCV-1 capsid has been deposited in the Protein Data Bank (PDB) under the accession number 8H2I. The updated gene sequences of *a533r* and *a383r* have been deposited at GenBank under the accession numbers AAC96900.1 and AAC96750.2, respectively.
