# Supplementary material for: Near-atomic, non-icosahedrally averaged structure of giant virus Paramecium bursaria chlorella virus 1
Source: Nat Commun. 2022 Oct 29;13:6476. doi: 10.1038/s41467-022-34218-4 (PMC9617893; doi:10.1038/s41467-022-34218-4)
Supplement: Supplementary file 1 — Supplementary Information [file 41467_2022_34218_MOESM1_ESM.pdf]

Supplementary Informations for

Near-atomic, non-icosahedrally averaged structure of giant virus Paramecium bursaria chlorella virus 1

Qianqian Shao, Irina V. Agarkova, Eric A. Noel, David D. Dunigan, Yunshu Liu, Aohan Wang, Mingcheng Guo, Linlin Xie, Xinyue Zhao, Michael G. Rossmann, James L. Van Etten, Thomas Klose, Qianglin Fang

This PDF file includes:

Supplementary Figs. 1 to 9

Supplementary Tables 1 to 6

Supplementary References

Other Supplementary Informations for this manuscript include the following:

Supplementary Movies 1 to 2

Supplementary Figures

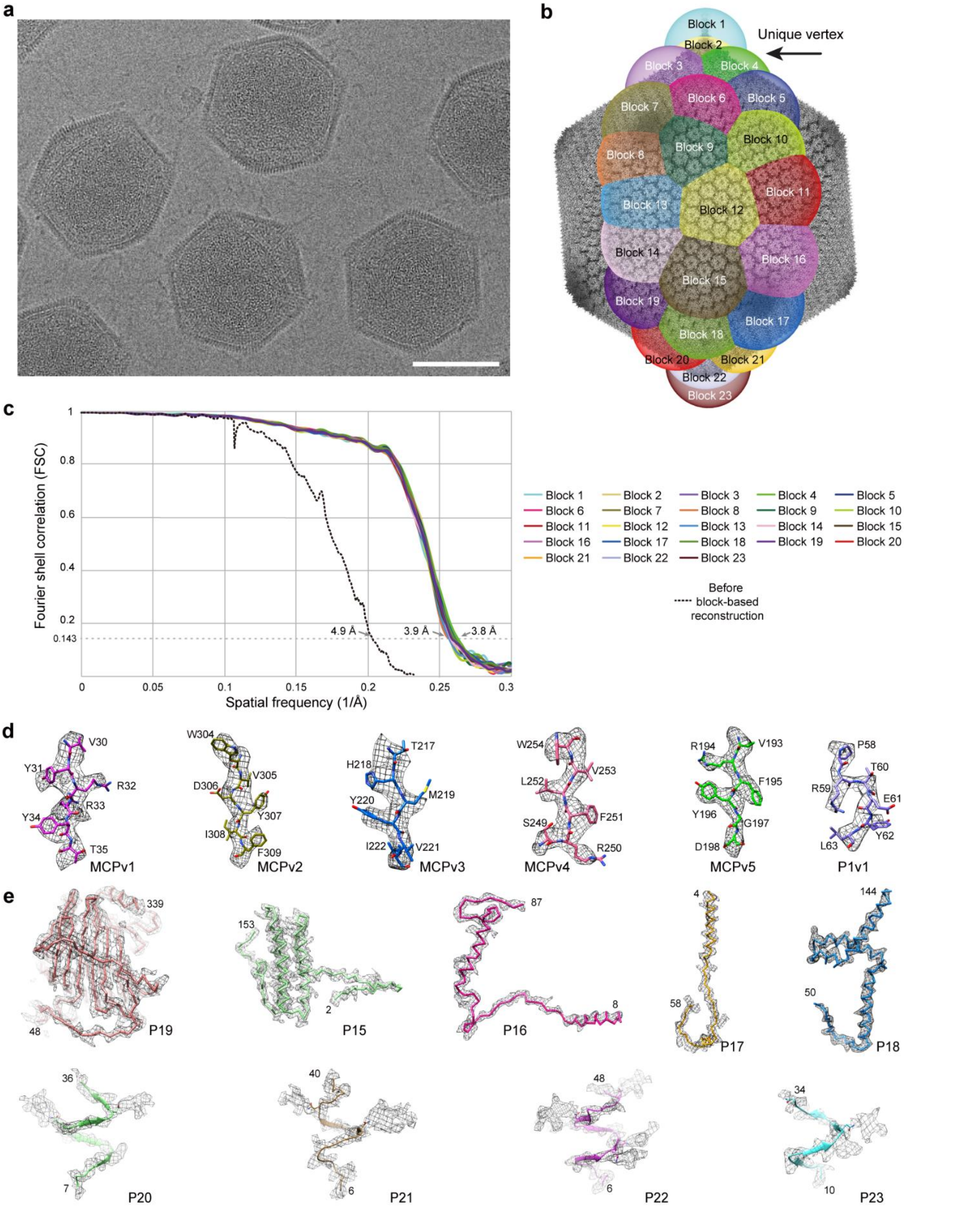

Supplementary Figure 1 | Resolution estimation and cryo-EM densities of the previously unresolved proteins.

a, Representative cryo-EM micrograph of PBCV-1. Scale bar = 100 nm. **b**, Block distribution within one five-fold asymmetric unit of the five-fold averaged, cryo-EM map of PBCV-1. **c**, FSC curves of each block and the cryo-EM map before applying the “block-based” reconstruction method calculated based on the FSC 0.143 cut-off. **d**, Cryo-EM densities of representative regions of the variants of the MCP and the penton protein, superposed with their atomic models. **e**, Cryo-EM densities of the previously unresolved minor capsid proteins, superposed with their atomic models.

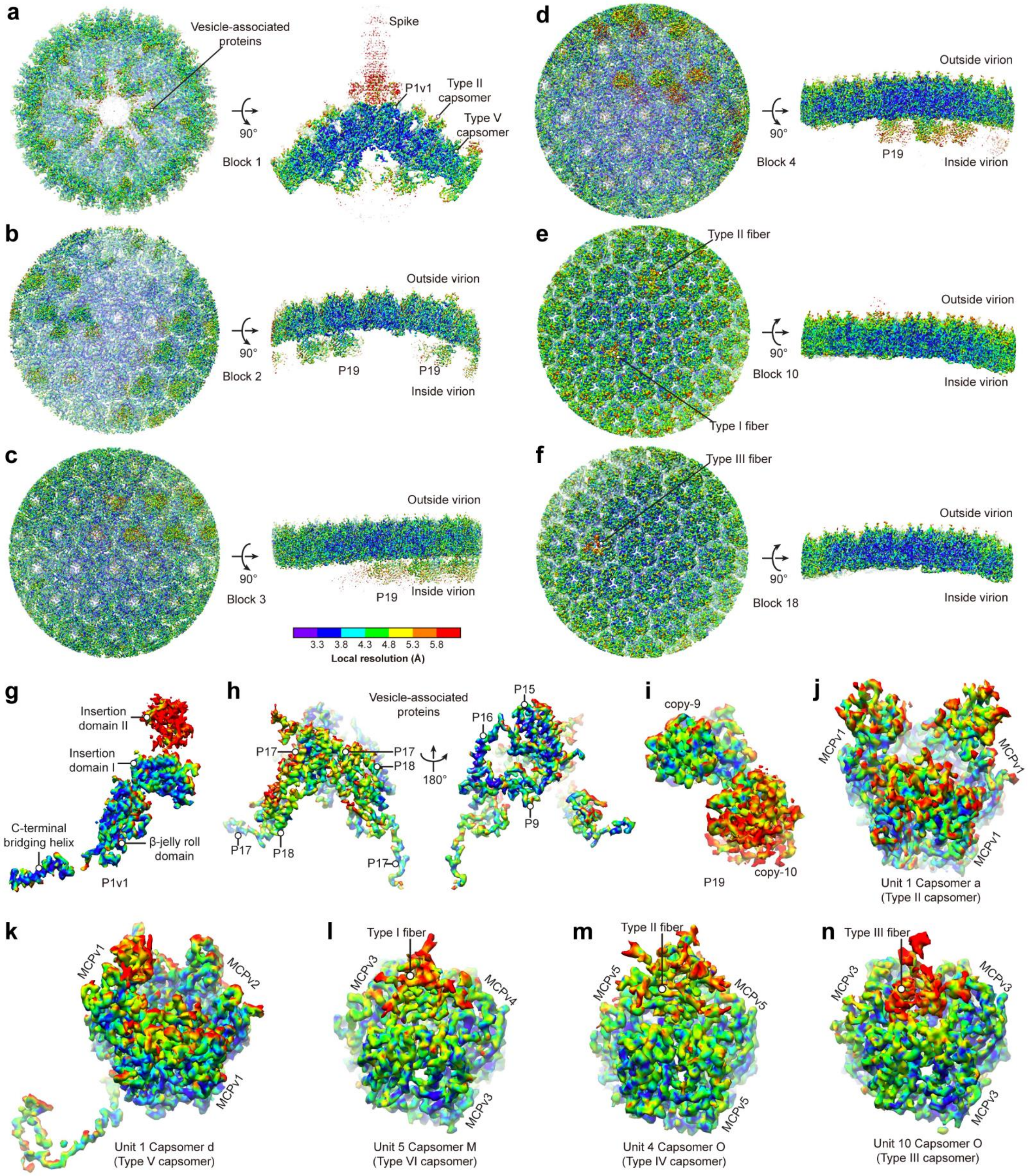

Supplementary Figure 2 | Local resolution estimation of the cryo-EM maps.

Local resolution maps of block 1 (**a**), block 2 (**b**), block 3 (**c**), block 4 (**d**), block 10 (**e**) and block 18 (**f**), P1v1 (**g**), vesicle-associated proteins (**h**), two copies of P19 (**i**), type II pseudo-hexameric capsomer (**j**), type V pseudo-hexameric capsomer (**k**), type I fiber and type VI pseudo-hexameric capsomer (**l**), type II fiber and type IV pseudo-hexameric capsomer (**m**), and type III fiber and type III pseudo-hexameric capsomer (**n**), as estimated using RESMAP[1].

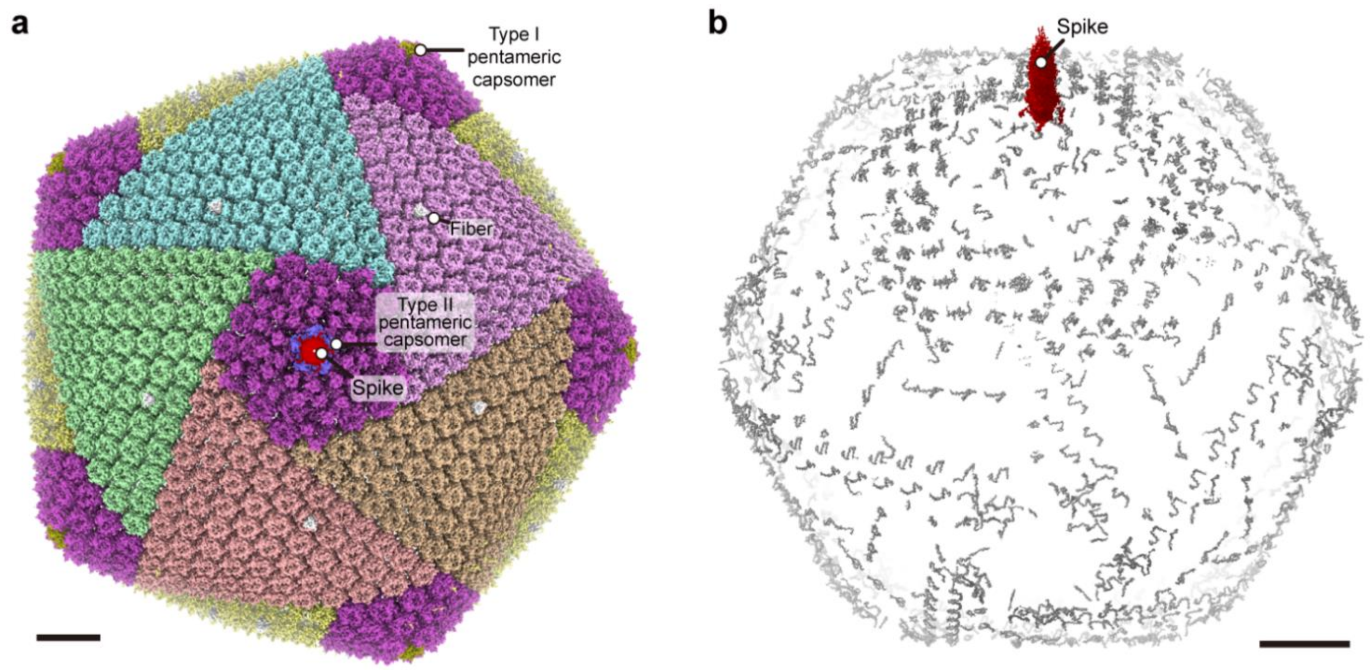

Supplementary Figure 3 | Symmetrons and uninterpreted densities of the five-fold averaged cryo-EM map of PBCV-1. a, Trisymmetrons and pentasymmetrons in the five-fold averaged cryo-EM map of PBCV-1, looking down the spike in the unique vertex. **b**, Uninterpreted densities of the five-fold averaged cryo-EM map of PBCV-1. Scale bar = 20 nm.

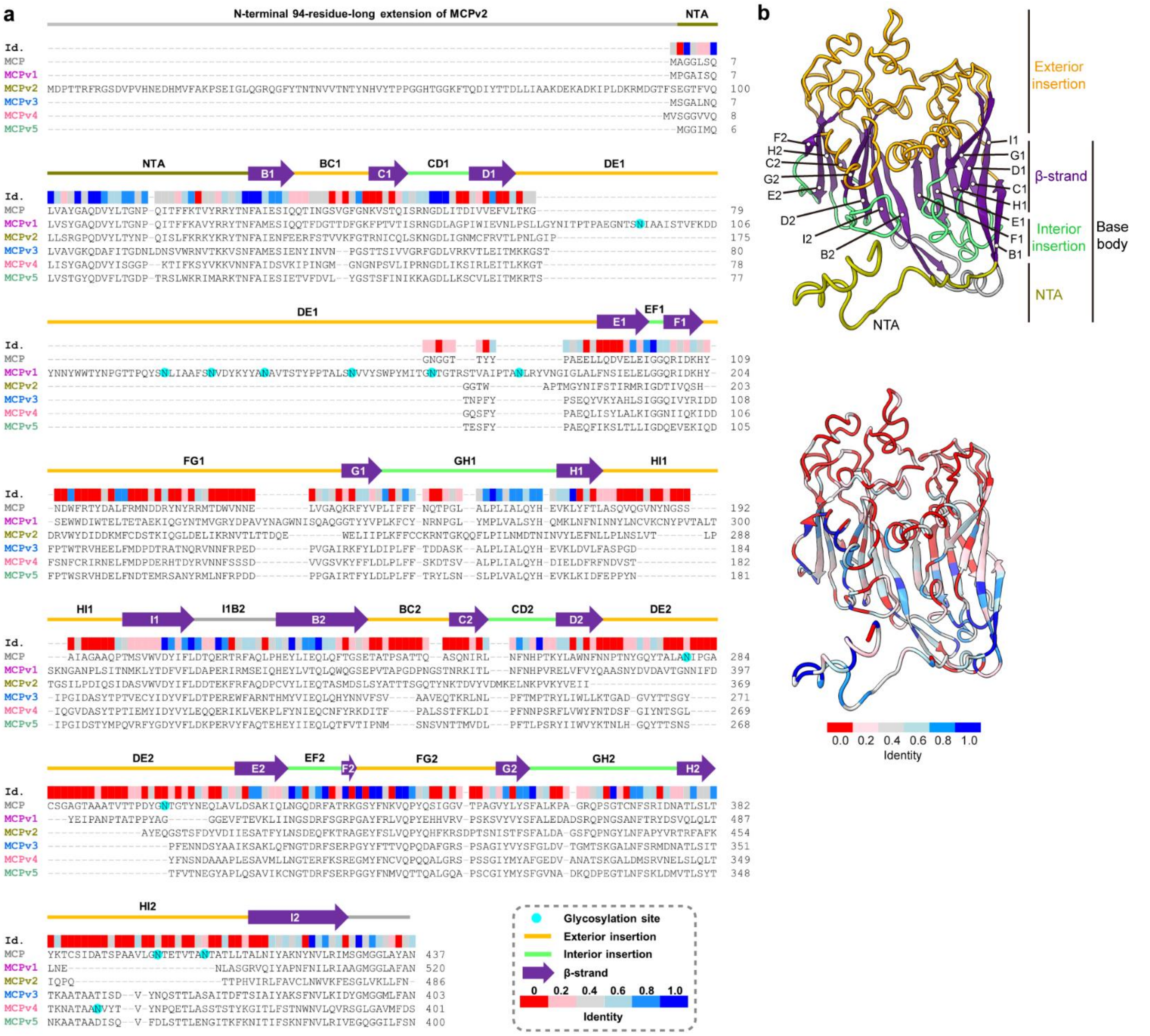

Supplementary Figure 4 | Sequence alignment of MCP and its variants.

a, sequence alignment of MCP and its variants. The identity values for each residue in the MCP sequence were calculated by comparing that residue of the MCP with corresponding residues of the MCP variants (1 being conserved, and 0 being not conserved). **b**, Ribbon diagrams of one MCP molecule showing different structural regions (up) and identity values as indicated in **a** (down).

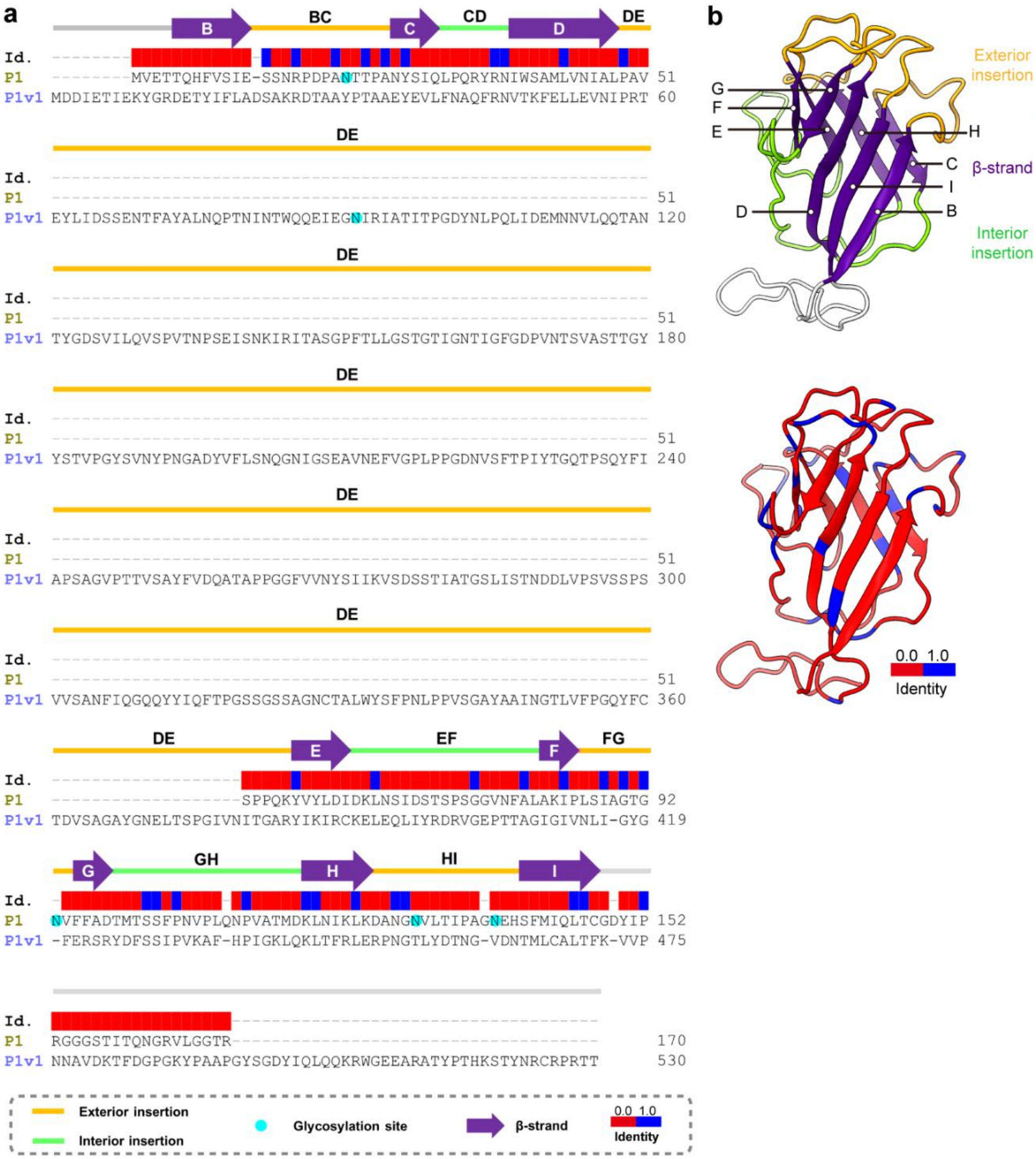

Supplementary Figure 5 | Sequence alignment of P1 and P1v1.

a, Sequence alignment of the penton protein P1 and the penton protein variant P1v1. The identity values were calculated similarly as in Supplementary Fig. 4a. **b**, Ribbon diagrams of one P1 molecule showing the structural regions (up) and identity values as indicated in **a** (down).

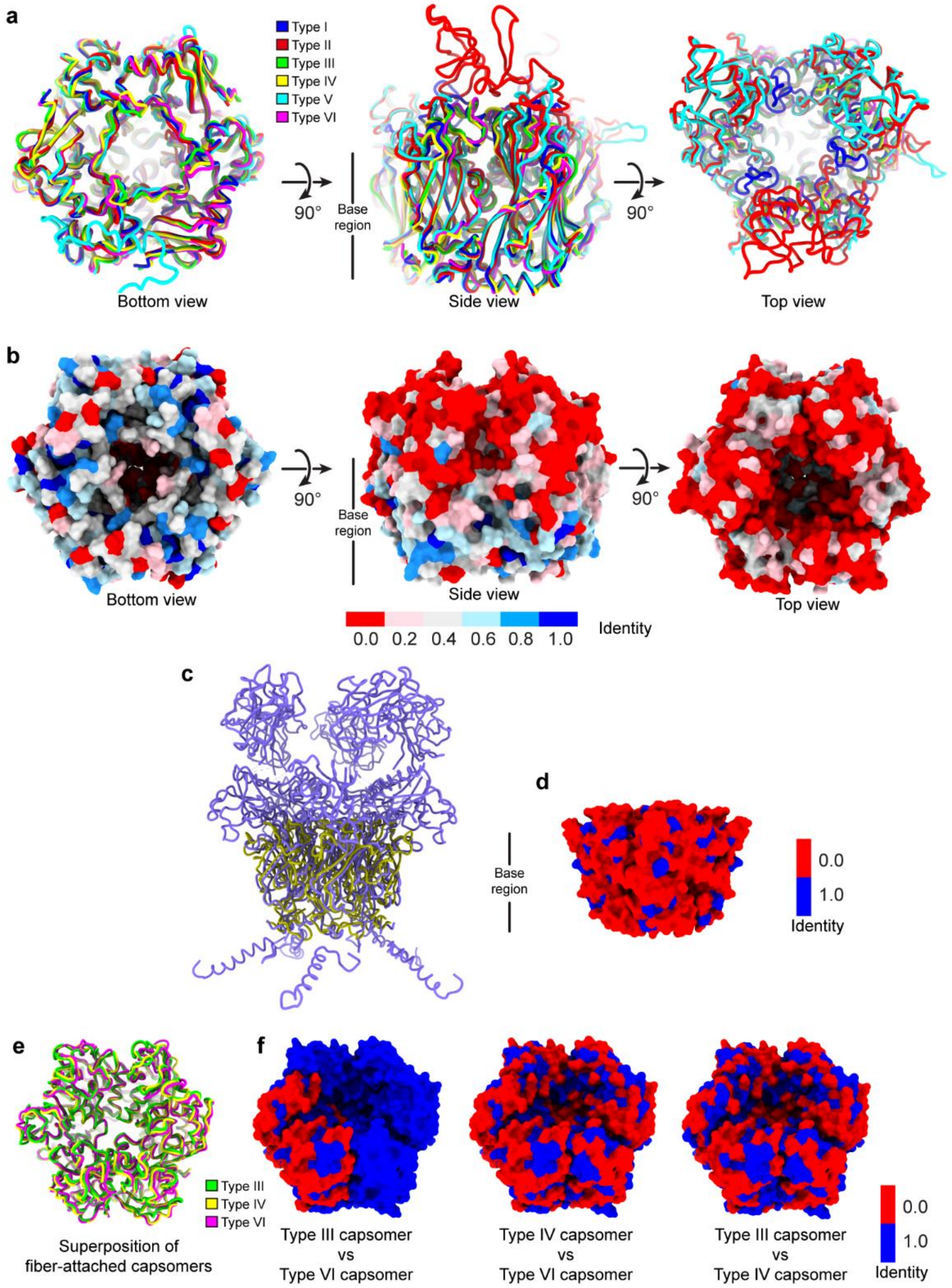

Supplementary Figure 6 | Structural comparisons and sequence identity analyses between pseudo-hexameric and between pentameric capsomers.

a, Backbone superposition of all the six types of pseudo-hexameric capsomers. **b**, Surface identity between the MCP and all the MCP variants. The identity values were calculated in the same way as in Supplementary Fig. 4a. **c**, Backbone superposition of the type I and type II pentameric capsomers. **d**, Surface identity between the type I and type II pentameric capsomers. The identity values were calculated using the same way as in Supplementary Fig. 5a. **e**, Backbone superposition of the three types of fiber-attached capsomers. **f**, Surface identities between any two types of fiber-attached capsomers. The identity values were calculated similarly as in Supplementary Fig. 4a.

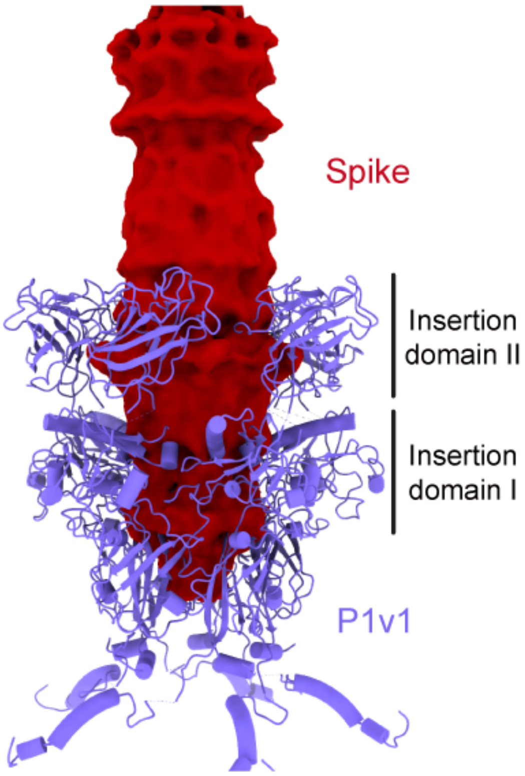

Supplementary Figure 7 | Association between the spike and type II pentameric capsomer.
The structures of the spike (dark red) and the type II pentameric capsomer (cornflower blue) are shown as cryo-EM density map and ribbon models, respectively.

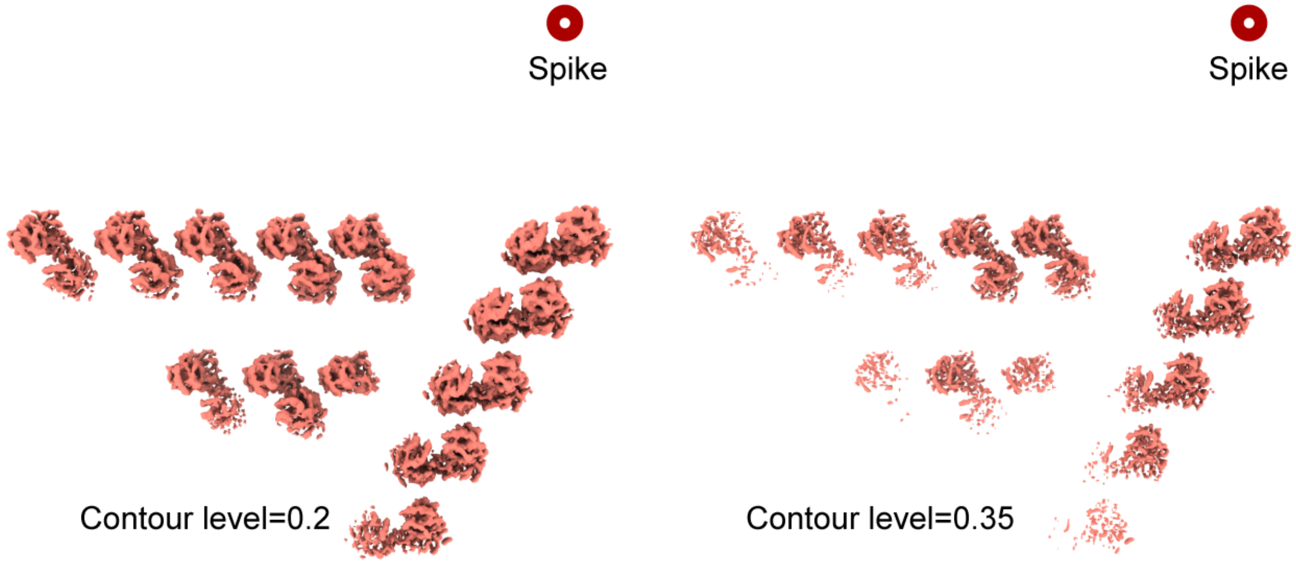

Supplementary Figure 8 | Cryo-EM densities of the 25 copies of the finger proteins displayed with two contour levels.

A dark red circle is used to indicate the position of the viral spike in the unique vertex.

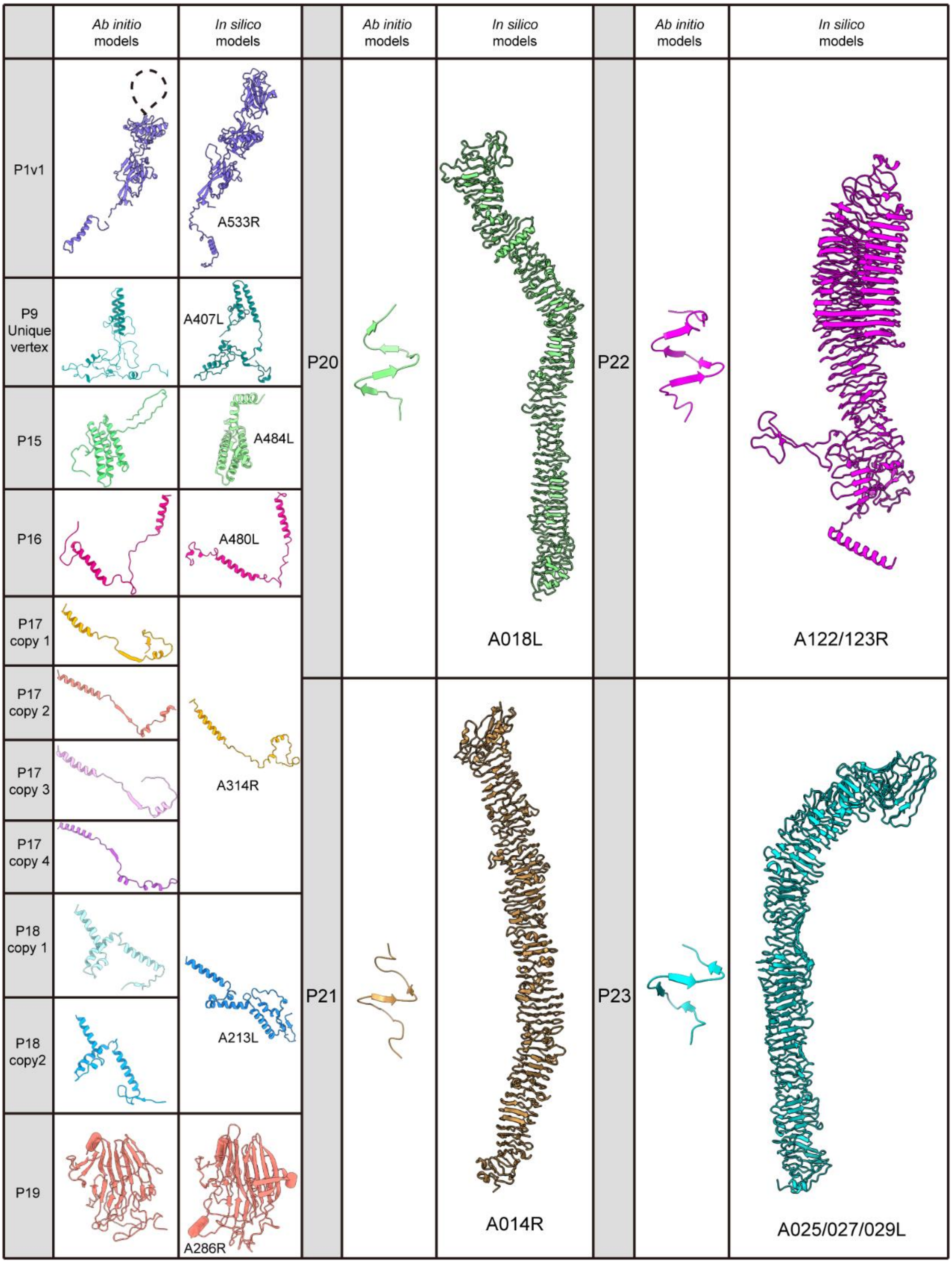

Supplementary Figure 9 | Structure comparisons between the *ab initio* models and their *in silico* model candidates.

The *ab initio* models and the *in silico* models were manually built using the cryo-EM density maps and computed using AlphaFold2[2] and/or RoseTTAFold[3], respectively.

Supplementary Tables

Supplementary Table 1 | Information on identified proteins.

Protein name	Gene name	Icosahedral asymmetric units where this protein is present	Copy number within one virion
MCP	*a430l*	All	4,905
MCPv1 [a]	*a622l*	1	40
MCPv2 [a]	*a383r*	1	5
MCPv3 [a]	*a011l*	1, 5, 7, 10, 11	55
MCPv4 [a]	*a010r*	1, 5, 7, 11	20
MCPv5 [a]	*a558l*	4	15
P1	*a310l*	2–12	55
P1v1 [a]	*a533r*	1	5
P2	*a342l*	All	60
P3	*a523r*	All	180
P4	*a572r*	All	240
P5	*a526r*	All	60
P6	*a203r*	All	60
P7	*a262/263l*	2–12	55
P8	*a644r*	All	60
P9	*a407l*	All	60
P10	*a454l*	2–12	110
P11	*a352l*	All	720
P12	*a139l*	All	60
P13	*a421r*	All	60
P14	*a500l*	2–12	55
P15 [a]	*a484l*	1	5
P16 [a]	*a480l*	1	5
P17 [a]	*a314r*	1	20
P18 [a]	*a213l*	1	10
P19 [a]	*a286r*	1–3	125
P20 [a]	*a018l*	1, 5, 7, 11	40
P21 [a]	*a014r*	1, 5, 7, 11	20
P22 [a]	*a122/123r*	4	15
P23 [a]	*a025/027/029l*	10	15

[a] Previously unresolved proteins.

Supplementary Table 2 | Model validation statistics on previously unresolved proteins.

Protein name	Protein sequence length	Chain ID[a]	Ordered part	CC[b]	Resolution (FSC=0.5)[c]	Resolution (FSC=0.25)[c]
MCPv1	520	bu	2–518	0.83	4.1	3.8
		bv	2–518	0.80	4.1	3.8
		bw	2–518	0.81	4.1	3.7
		bx	5–518	0.84	4.1	3.7
		by	2–518	0.83	4.1	3.7
		bz	11–518	0.80	4.2	3.8
		bA	2–517	0.82	4.2	3.7
		bB	2–518	0.81	4.2	3.9
MCPv2	486	bC	2–486	0.73	4.2	3.8
MCPv3	403	dd	3–403	0.82	4.1	3.8
		dp	3–403	0.82	4.1	3.8
		dr	3–403	0.81	4.1	3.8
		df	3–403	0.82	4.2	3.8
		dj	3–403	0.80	4.2	3.8
		bD	3–403	0.82	4.2	3.8
		dl	3–403	0.77	4.2	3.9
		cZ	3–403	0.80	4.2	3.8
		dk	3–403	0.80	4.2	3.9
		bF	3–403	0.81	4.3	3.9
		cX	3–403	0.79	4.3	3.9
MCPv4	401	dq	3–399	0.83	4.2	3.8
		de	3–399	0.83	4.2	3.9
		bE	3–399	0.82	4.2	3.9
		cY	3–399	0.79	4.3	3.9
MCPv5	400	cR	2–400	0.81	4.2	3.8
		cT	2–400	0.82	4.2	3.9
		cS	2–400	0.81	4.3	3.9
P1v1	530	bG	2–203, 210–367, 370–479, 491–524	0.67	4.3	3.8
P15	155	ch	2–153	0.74	4.1	3.8
P16	93	ci	8–87	0.72	4.5	3.5
P17	80	ck	2–58	0.75	4.3	3.6
		cm	5–67	0.74	4.4	3.7
		cj	5–63	0.74	4.4	3.6
		cl	4–58	0.74	4.5	3.8
P18	148	cn	49–146	0.76	4.1	3.6
		co	50–144	075	4.2	3.6
P19	378	cv	48–339	0.74	4.4	4.0
		cx	48–339	0.74	4.4	4.1
		cL	48–339	0.74	4.5	4.1
		ct	48–339	0.76	4.6	4.2
		cJ	48–339	0.76	4.6	4.2
		cw	60–339	0.73	4.6	4.2
		cH	48–339	0.75	4.6	4.2
		cF	48–339	0.75	4.7	4.2

		cr	48–339	0.75	4.7	4.3
		cp	48–339	0.73	4.7	4.2
		cB	48–339	0.74	4.7	4.2
		cy	60–339	0.76	6.8	4.3
		cD	48–339	0.73	7.0	4.3
		cE	60–339	0.75	7.1	4.4
		cN	48–339	0.7	7.5	4.3
		cI	61–339	0.75	7.7	4.4
		cu	60–339	0.73	7.9	4.5
		cC	61–339	0.72	7.9	4.3
		cG	58–339	0.74	7.9	4.5
		cK	61–339	0.72	7.9	4.6
		cq	58–339	0.69	8.1	4.6
		cz	48–339	0.69	8.1	4.4
		cs	65–339	0.75	8.4	4.5
		cA	61–339	0.62	8.6	6.9
		cM	63–339	0.66	8.6	5.7
P20	1335	da	6–37	0.81	4.1	3.4
		dc	7–34	0.83	4.2	3.4
		cO	7–36	0.81	4.3	3.5
		ds	6–37	0.80	4.3	3.8
		du	7–36	0.75	4.3	3.5
		cQ	7–37	0.81	4.3	3.4
		dg	6–37	0.79	4.4	3.6
		di	7–35	0.76	4.4	3.6
P21	1369	cP	6–40	0.80	4.2	3.5
		dh	6–40	0.79	4.3	3.7
		dt	6–40	0.79	4.4	3.6
		db	7–36	0.79	4.5	3.6
P22	1343	cU	6–48	0.79	4.3	3.9
		cV	6–48	0.78	4.3	3.9
		cW	6–48	0.78	4.4	3.6
P23	1359	dm	10–34	0.65	4.5	3.7
		dn	10–34	0.68	4.5	3.3
		do	10–34	0.59	4.9	3.6

[a] Chain ID in the coordinate file (PDB ID: 8H2I).

[b] Real-space correlation coefficient between the atomic models and their density maps.

[c] Model-to-map FSCs between the atomic models and their density maps.

Supplementary Table 3 | Protein sequence identities between MCP, P1 and their variants.

Variants	Protein sequence identity between this variant with MCP
MCPv1	42.8%
MCPv2	28.1%
MCPv3	38.0%
MCPv4	35.5%
MCPv5	36.8%
	**Protein sequence identity between this variant with P1**
P1v1	10.6%

The protein sequence identity is calculated according to the ratio of the number of matched residues to the sequence length of MCP or P1.

Supplementary Table 4 | Information on identified glycosylation sites.

Protein	Glycosylation sites
MCP	$AN_{280}IP$
	$GN_{302}TG$
	$GN_{399}TE$
	$AN_{406}TA$
MCPv1	$SN_{95}IA$
	$SN_{124}LI$
	$SN_{131}VD$
	$AN_{139}AV$
	$SN_{152}VV$
	$GN_{164}TG$
	$AN_{177}LR$
MCPv4	$AN_{357}VY$
P1	$AN_{21}TT$
	$GN_{93}VF$
	$GN_{129}VL$
	$GN_{137}EH$
P1v1	$GN_{91}IR$
P20	$GN_{22}LS$
	$GN_{31}GA$
P21	$GN_{21}LV$
	$GN_{32}GG$
P22	$GN_{23}IA$
	$AN_{26}VI$
	$DN_{31}GN$
	$GN_{33}VI$
	$GN_{46}GA$
P23	$GN_{24}VF$
	$GN_{33}AS$

Supplementary Table 5 | Primers used in this work.

**PBCV-1 Gene Primer**	**Sequence**
PBCV-1 *a533r* F Primer	5’-GCGCAAACTTTATTCAAGGAC-3’
PBCV-1 *a533r* R Primer	5’-TAATCCCCAGAGTATCCAGGG-3’
PBCV-1 *a383r* F Primer	5’-CTCGGAGAAATCTTCTCGC-3’
PBCV-1 *a383r* F Primer (2)	5’-CGGATGTTTACGTTGACATG-3’
PBCV-1 *a383r* R Primer	5’-CTAAACCCGCACCTAAACC-3’

Supplementary Table 6 | Cryo-EM data collection, refinement and validation statistics.

**Map**	PBCV-1 C5<br>(EMD-34438, PDB: 8H2I)
**Data collection and processing**	
Microscope	FEI Titan Krios
Magnification	18,000
Voltage (kV)	300
Detector	Gatan K2 Summit detector
Recording mode	Super-resolution or counting mode
Dose rate (e^-/(pixel·s))	~8
Frame exposure time (ms)	200
Movie micrograph exposure time (s)	~8
Total dose (e^-/Å^2)	~24
Defocus range (μm)	1.0–4.0
Pixel size (Å)	1.62 (physical pixel size)
Symmetry imposed	C5
Map resolution (Å)	3.8–3.9
FSC threshold	0.143
Map sharpening *B*-factor (Å^2)	-120
**Refinement**	
Initial model used (PDB code)	6NCL
R.m.s. deviations	
Bond lengths (Å)	0.010
Bond angles (°)	1.43
Validation	
MolProbity score	1.37
Clashscore	2.49
Poor rotamers (%)	0.14
Ramachandran plot	
Favored (%)	95.22
Allowed (%)	4.40
Disallowed (%)	0.38

Supplementary References

1 Kucukelbir, A., Sigworth, F. J. & Tagare, H. D. Quantifying the local resolution of cryo-EM density maps. *Nat. Methods* **11**, 63–65 (2014).

2 Jumper, J. *et al.* Highly accurate protein structure prediction with AlphaFold. *Nature* **596**, 583–589 (2021).

3 Baek, M. *et al.* Accurate prediction of protein structures and interactions using a three-track neural network. *Science* **373**, 871-876 (2021).
