# Supplementary material for: Near-atomic, non-icosahedrally averaged structure of giant virus Paramecium bursaria chlorella virus 1
Source: Nat Commun. 2022 Oct 29;13:6476. doi: 10.1038/s41467-022-34218-4 (PMC9617893; doi:10.1038/s41467-022-34218-4)
Supplement: Supplementary file 2 — Description of Additional Supplementary Files [file 41467_2022_34218_MOESM2_ESM.pdf]

Description of Additional Supplementary Files

Supplementary Movie 1.

Overall structure of the outer capsid shell of PBCV-1.

Supplementary Movie 2.

The minor capsid protein layer below the outer capsid shell of PBCV-1.
