# Supplementary material for: Near-atomic, non-icosahedrally averaged structure of giant virus Paramecium bursaria chlorella virus 1
Source: Nat Commun. 2022 Oct 29;13:6476. doi: 10.1038/s41467-022-34218-4 (PMC9617893; doi:10.1038/s41467-022-34218-4)
Supplement: Supplementary file 5 — Reporting Summary [file 41467_2022_34218_MOESM5_ESM.pdf]

nature portfolio

Corresponding author(s): Qianglin Fang, Thomas Klose and James L. Van Etten

Last updated by author(s): Oct 6, 2022

Reporting Summary

Nature Portfolio wishes to improve the reproducibility of the work that we publish. This form provides structure for consistency and transparency in reporting. For further information on Nature Portfolio policies, see our Editorial Policies and the Editorial Policy Checklist.

Statistics

For all statistical analyses, confirm that the following items are present in the figure legend, table legend, main text, or Methods section.

n/a	Confirmed	
☒	☐	The exact sample size (*n*) for each experimental group/condition, given as a discrete number and unit of measurement
☒	☐	A statement on whether measurements were taken from distinct samples or whether the same sample was measured repeatedly
☒	☐	The statistical test(s) used AND whether they are one- or two-sided<br>*Only common tests should be described solely by name; describe more complex techniques in the Methods section.*
☒	☐	A description of all covariates tested
☒	☐	A description of any assumptions or corrections, such as tests of normality and adjustment for multiple comparisons
☒	☐	A full description of the statistical parameters including central tendency (e.g. means) or other basic estimates (e.g. regression coefficient) AND variation (e.g. standard deviation) or associated estimates of uncertainty (e.g. confidence intervals)
☒	☐	For null hypothesis testing, the test statistic (e.g. *F*, *t*, *r*) with confidence intervals, effect sizes, degrees of freedom and *P* value noted<br>*Give P values as exact values whenever suitable.*
☒	☐	For Bayesian analysis, information on the choice of priors and Markov chain Monte Carlo settings
☒	☐	For hierarchical and complex designs, identification of the appropriate level for tests and full reporting of outcomes
☒	☐	Estimates of effect sizes (e.g. Cohen's *d*, Pearson's *r*), indicating how they were calculated

Our web collection on statistics for biologists contains articles on many of the points above.

Software and code

Policy information about availability of computer code

Data collection	Leginon 3.2
Data analysis	MotionCorr 2, CTFFIND 4, Appion 3.2, Gautomatch 0.53, Relion 1.4, jspr 2.21a, Coot 0.7.1, RoseTTAFold, AlphaFold2.0, TMHMM 2.0, HMMTOP 2.0, Phobius, Phenix 1.17, Rosetta 3, Dali search (web server), RESMAP 1.1.4, Chimera 1.13.1rc, ChimeraX 1.3

For manuscripts utilizing custom algorithms or software that are central to the research but not yet described in published literature, software must be made available to editors and reviewers. We strongly encourage code deposition in a community repository (e.g. GitHub). See the Nature Portfolio guidelines for submitting code & software for further information.

Data

Policy information about availability of data

All manuscripts must include a data availability statement. This statement should provide the following information, where applicable:

- Accession codes, unique identifiers, or web links for publicly available datasets
- A description of any restrictions on data availability
- For clinical datasets or third party data, please ensure that the statement adheres to our policy

The five-fold averaged cryo-EM reconstruction before and after applying the “block-based” reconstruction method of PBCV-1 have been deposited in the Electron Microscopy Data Bank (EMDB) under the accession number EMD-34439 and EMD-34438, respectively. The atomic model of the unique vertex and newly resolved previously unresolved polypeptide chains in the other regions of the PBCV-1 capsid have been deposited in the Protein Data Bank (PDB) under the accession

number 8H2I, respectively. The updated gene sequences of a533r and a383r have been deposited at GenBank under the accession numbers AAC96900.1 and AAC96750.2, respectively.

Human research participants

Policy information about studies involving human research participants and Sex and Gender in Research.

Reporting on sex and gender	Not applicable.
Population characteristics	Not applicable.
Recruitment	Not applicable.
Ethics oversight	Not applicable.

Note that full information on the approval of the study protocol must also be provided in the manuscript.

Field-specific reporting

Please select the one below that is the best fit for your research. If you are not sure, read the appropriate sections before making your selection.

☒ Life sciences ☐ Behavioural & social sciences ☐ Ecological, evolutionary & environmental sciences

For a reference copy of the document with all sections, see nature.com/documents/nr-reporting-summary-flat.pdf

Life sciences study design

All studies must disclose on these points even when the disclosure is negative.

Sample size	A total of ~16,200 movie stacks were automatically collected using the Leginon program. These samples (data) turned out to be sufficient for us to extend the resolution of the cryo-EM reconstruction to near-atomic level.
Data exclusions	No data was excluded.
Replication	The cryo-EM structure reported in this study is consistent with the previously determined structure. All attempts at replication were successful.
Randomization	Randomization is not applicable to the study with automated movie acquisition and data analysis being conducted using mathematical and unbiased (reference-free) class averaging.
Blinding	Blinding is not applicable to the Cryo-EM data acquisition and data processing as the identity of the sample is required towards determining a de novo structure.

Reporting for specific materials, systems and methods

We require information from authors about some types of materials, experimental systems and methods used in many studies. Here, indicate whether each material, system or method listed is relevant to your study. If you are not sure if a list item applies to your research, read the appropriate section before selecting a response.

Materials & experimental systems

n/a	Involved in the study	
☒	☐	Antibodies
☒	☐	Eukaryotic cell lines
☒	☐	Palaeontology and archaeology
☒	☐	Animals and other organisms
☒	☐	Clinical data
☒	☐	Dual use research of concern

Methods

n/a	Involved in the study	
☒	☐	ChIP-seq
☒	☐	Flow cytometry
☒	☐	MRI-based neuroimaging
